# Influence of the Molecular Weight and the Presence of Calcium Ions on the Molecular Interaction of Hyaluronan and DPPC

**DOI:** 10.3390/molecules25173907

**Published:** 2020-08-27

**Authors:** Thomas Zander, Vasil M. Garamus, Andra Dédinaité, Per M. Claesson, Piotr Bełdowski, Krzysztof Górny, Zbigniew Dendzik, D. C. Florian Wieland, Regine Willumeit-Römer

**Affiliations:** 1 Institute of Materials Research, Helmholtz-Zentrum Geesthacht: Centre for Materials and Costal Research, Max-Planck-Straße 1, 21502 Geesthacht, Germany; thomas.zander@hzg.de (T.Z.); vasyl.haramus@hzg.de (V.M.G.); regine.willumeit@hzg.de (R.W.-R.); 2 Department of Chemistry, Surface and Corrosion Science, School of Engineering Sciences in Chemistry, Biotechnology and Health, KTH Royal Institute of Technology, Drottning Kristinas väg 51, SE-10044 Stockholm, Sweden; andra@kth.se (A.D.); percl@kth.se (P.M.C.); 3 Division of Bioscience and Materials, RISE Research Institutes of Sweden, SE-114 86 Stockholm, Sweden; 4 Institue of Mathematics and Physics, UTP University of Science and Technology, al. Kaliskiego 7, 85-796 Bydgoszcz, Poland; piobel000@utp.edu.pl; 5 Faculty of Science and Technology, University of Silesia in Katowice, 75 Pułku Piechoty 1A, 41-500 Chorzów, Poland; krzysztof.gorny@us.edu.pl (K.G.); dendzik@us.edu.pl (Z.D.)

**Keywords:** phospholipid, hyaluronan, molecular interaction, phase behavior, binding distribution

## Abstract

Hyaluronan is an essential physiological bio macromolecule with different functions. One prominent area is the synovial fluid which exhibits remarkable lubrication properties. However, the synovial fluid is a multi-component system where different macromolecules interact in a synergetic fashion. Within this study we focus on the interaction of hyaluronan and phospholipids, which are thought to play a key role for lubrication. We investigate how the interactions and the association structures formed by hyaluronan (HA) and 1,2-dipalmitoyl-sn-glycero-3-phosphocholine (DPPC) are influenced by the molecular weight of the bio polymer and the ionic composition of the solution. We combine techniques allowing us to investigate the phase behavior of lipids (differential scanning calorimetry, zeta potential and electrophoretic mobility) with structural investigation (dynamic light scattering, small angle scattering) and theoretical simulations (molecular dynamics). The interaction of hyaluronan and phospholipids depends on the molecular weight, where hyaluronan with lower molecular weight has the strongest interaction. Furthermore, the interaction is increased by the presence of calcium ions. Our simulations show that calcium ions are located close to the carboxylate groups of HA and, by this, reduce the number of formed hydrogen bonds between HA and DPPC. The observed change in the DPPC phase behavior can be attributed to a local charge inversion by calcium ions binding to the carboxylate groups as the binding distribution of hyaluronan and 1,2-dipalmitoyl-sn-glycero-3-phosphocholine is not changed.

## 1. Introduction

Hyaluronan (HA) is one of the important physiological bio macromolecules which is ubiquitous in the body. It is a high molecular weight anionic and linear polysaccharide consisting of repeating disaccharide units of β-1,3-d-glucuronic acid and β-1,4-*N*-acetyl-d-glucosamine. The pKa of the monomer d-glucuronic acid and the polymer has been estimated to be 3.23 and 2.9, respectively, and the distance between adjacent charges along the polymer chain is about 1 nm [1]. Hyaluronan is regarded as a wormlike polyelectrolyte with an intrinsic persistence length ranging from 4.5 to 9 nm in high ionic strength solutions [2]. The role of hyaluronan is quite diverse, ranging from being important for cell proliferation, cell migration, the connective tissue and the synovial fluid. The latter one is a multi-component aqueous system that contains, in addition to hyaluronan, a complex mixture of phospholipids, proteins, proteoglycans and ions [3,4]. The reason for the interest in the working mechanisms of the synovial joints is due to their excellent lubrication properties with friction coefficients as low as 0.002–0.006 under high shear and load conditions [3,5]. The secret of this performance is thought to be a fluid water layer in between the sliding surfaces and friction reduction occurs via hydration repulsion generated by water interacting with biomolecules adsorbed from the synovial fluid to the cartilage surface, e.g., lubricin, phospholipids and HA [4,6,7,8,9]. Different investigations suggest that not only one component but rather a mixture of specific constitutes has to be present to allow for the exceptional good lubrication properties in joints as, for example, the combined presence of albumin, γ-globulin, DPPC and HA leads to reduction by a factor of 4 compared solutions with the single components [10,11]. One suggestion is that the sliding occurs via boundary lubrication in between multilayer phospholipid structures, which form at the cartilage surface [3,12]. The important sliding plane seems to be in between the phospholipid head group structures where a thin layer of water is maintained [12,13,14]. The question arises how the association structures adapt to stable well-defined structures that promote a bond but yet retain a fluid and easily sheared layer of water under high loads and shear conditions.

Among the different components in the synovial fluid, hyaluronan and phospholipids are thought to be of major importance for lubrication. This view arises from the fact that phospholipids are very efficient in reducing boundary friction [15,16,17]. On the other hand, hyaluronan has been shown to be able to relieve osteoarthritis and degenerated articular cartilage by injection into the knee [18]. The effect is mainly due to its ability to control the viscosity of the synovial fluid and retain water [5]. Hydrophobic regions representing repeating structures of eight CH groups along the HA chains have been identified [19], and these hydrophobic sites are perceived as being important for network formation and aggregation of HA [20]. HA is able to reduce friction on its own to some extent, and it is important for the rheological properties of the synovial fluid as it controls viscosity [10,21,22].

For these reasons the interaction of 1,2-dipalmitoyl-sn-glycero-3-phosphocholine (DPPC) and HA has been the scope of different theoretical and experimental investigation to reveal the associate structures formed, their interactions and their behavior under load. Experimental investigations have reported on the accumulation of HA in-between dipalmitoylphosphaditcholin bilayers [23] and adsorption of HA to DPPC bilayers [17,24,25,26,27]. On the other hand, the study performed by Herzog et al. using HA with a high molecular weight (MW) could not observe any adsorption of HA to DPPC [28]. This observation is not in objection to the former cited studies, as an investigation reporting on the effect of the MW on the interaction shows that HA with high MW has a weak interaction with DPPC [29]. The reason for this might be the intrinsic structure of HA, where low MW species may form more intermolecular interactions as opposed to high MW species where more intramolecular interactions are expected.

Both Na^+^ and Cl^−^ ions have been reported to bind to DPPC membrane bilayers. Since the binding of Cl^−^ is weaker than that of Na^+^, the DPPC bilayer in NaCl electrolyte is expected to have a net positive surface charge [30]. Investigations of ion-membrane interactions suggest that calcium ions can be found at low concentrations of 1.2 mM to 2.4 mM in the synovial fluid [31,32], and it is well-known that CaCl_2_ is able to alter the structure of DPPC bilayers [33,34,35,36,37,38,39] and the charge density of HA [40]. Na^+^ and Ca^2+^ ions bind to phospholipid head groups via coordination of the cations by three and four lipid carbonyl oxygens, respectively [41]. The stoichiometry between calcium ions and phospholipids has been considered in several studies, with somewhat conflicting results. For instance, for 1-Palmitoyl-2-oleoylphosphatidylchol, the lipid/Ca^2+^ ratio was reported to be 1:2 in 5 M CaCl_2_ [39]. A binding stoichiometry of Ca^2+^/DPPC of 1:1 has also been proposed, and the binding constants of Ca^2+^ and Na^+^ were reported to be 37 M^−1^ and 0.25 M^−1^, respectively, signifying significantly stronger binding of Ca^2+^ ions. Still, other binding stoichiometry have been reported by Disalvo et al., where the phospholipid/Ca^2+^ ratio of 2:1 was obtained for the inner monolayer and 1:1 for the outer monolayer [42]. The interaction between metal ions and phospholipids is also temperature dependent, and the data suggest stronger binding in the gel phase than in the fluid phase. Unlike many anionic biopolymers, hyaluronan does not precipitate at high Ca^2+^ concentrations, which is related to its strong water binding capacity. Small angle X-ray scattering (SAXS) measurements in the semi dilute regime also suggest minor effects of calcium ions [40]. However, anomalous SAXS measurements have shown that in mixed solutions containing Na^+^ and Ca^2+^ ions, the calcium ions are preferentially located next to the hyaluronan chain [40].

It has also been shown that low concentration of calcium ions increases the interaction between HA and DPPC [29,43]. It was speculated that a possible mechanism is a charge reversal of the phosphate group by calcium ions resulting in a positive charge of DPPC. Indeed, theoretical calculations confirm that calcium ions promote long-lasting interactions between the carboxylate group of HA and the phosphate group of the phospholipid by creating a bridging connection [44]. Further, a reinforcement of the HA network by the adsorption of phospholipids has been observed [45,46].

To gain understanding of the interactions of the synovial fluid under loading conditions is challenging as only a limited number of techniques allow exploration of this situation on the molecular level. Investigations on the phase behavior of solid supported HA–DPPC composite bilayers demonstrated that the phase behavior is changed in comparison to that of DPPC bilayers alone. The reports indicate that HA–DPPC bilayers exhibit an increased robustness against high hydrodynamic pressure as compared to DPPC bilayers alone, allowing for a reversible phase change [27,47]. Further, a decoupling of the two DPPC leaflets could be observed for low MW of HA in the presence of calcium ions.

This investigation aims to further elucidate the effects of calcium ions and molecular weight on the molecular interactions between HA and DPPC by a combination of simulations and experimental approaches. To this end, we utilized DPPC vesicles as here no solid–liquid interfaces are present, which simplifies the system. The samples were studied under near physiological conditions of 150 mM sodium chloride and with 10 mM calcium chloride added. In former studies [29] it was observed that the MW of HA had an impact on the phase behavior and bilayer structures of DPPC, therefore, the phase behavior of HA was investigated by differential scanning calorimetry (DSC) and dynamic light scattering (DLS) in the MW range from 10 kDa to 2500 kDa. Based on these results, we decided to use HA of 10 kDa and 1500 kDa for small angle neutron scattering (SANS) measurements as this study focused on the formed structures and the structural arrangement of DPPC and HA to elucidate if the observed changes in the DSC could be attributed to a changed phospholipid packing. The HA with an MW of 10 kDa was chosen as we expected a stronger effect due to the small size if it would penetrate the lipid bilayer than that achieved by HA with an MW of 1500 kDa. To further investigate the impact of Ca^2+^ ions on the HA–DPPC arrangement, SAXS measurements on vesicles were performed. The decision on the 250 kDa for these measurements was based on the other measurements as the impact for low MW of HA is stronger. However, the usage of a too short HA polymer chain would have made it difficult to see an adsorbed layer like that experienced in former studies [29]. The electrophoretic mobility measurements were performed to achieve an understanding of the Ca^2+^ binding to the vesicles and how this influences the association with HA, and in these measurements HA with sufficiently large molecular weight are needed, and our choice fell on 800 kDa. To obtain further insight on the molecular level all-atom molecular dynamics (MD), simulations were performed and used as an aide for interpreting the experimental findings. With the available computational resources, modeling long chains of HA, with atomic mass over dozens kDa at the atomic level, is very difficult, if not impossible. To model longer HA chains, coarse-grained MD simulations could be utilized, although they could be insufficient to obtain the necessary level of details [48].

## 2. Results

### 2.1. Electrophoretic Mobility

**Figure 1:**
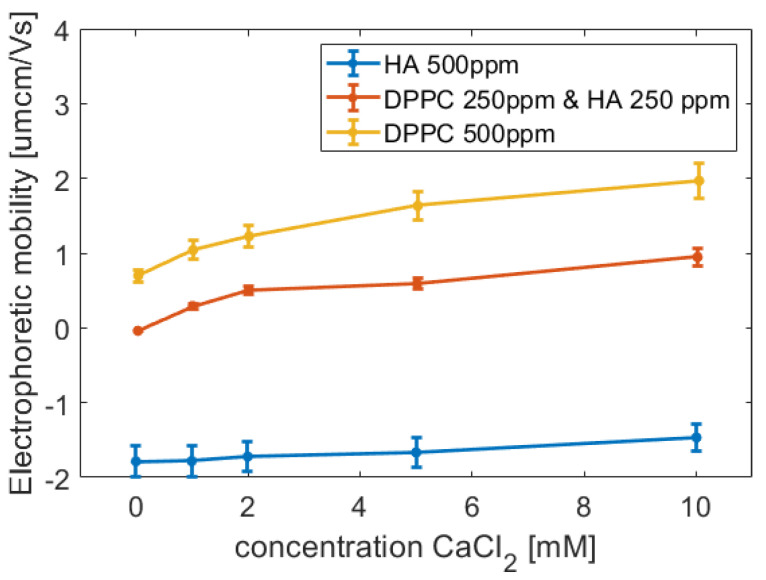
Electrophoretic mobility of DPPC vesicles, hyaluronan and their mixture as a function of CaCl_2_ concentration at 25 °C. All solutions contained 155 mM NaCl.

Figure 1 shows some data from electrophoretic mobility measurements. It can be observed that HA has a negative mobility, which is attributed to its anionic character. The electrophoretic mobility of hyaluronan became slightly less negative with increasing CaCl_2_ concentration, which suggests weak binding of Ca^2+^ ions. The positive electrophoretic mobility of DPPC vesicles was found to decrease in presence of hyaluronan, which is due to binding of hyaluronan to the DPPC vesicles. The decrease in electrophoretic mobility is due to the reduction in surface charge after adsorption of negatively charged HA and the increase in hydrodynamic diameter, from about 100 nm to about 140 nm, for these vesicles. Addition of calcium ions results in increased electrophoretic mobility for both DPPC vesicles alone and for DPPC vesicles decorated by hyaluronan. It appears that binding of hyaluronan to the DPPC vesicles has a rather low effect on the ability of calcium ions to bind to the DPPC headgroup, even though modelling has shown that calcium mediated ion bridges form between the phosphate group of DPPC and the carboxylate group of HA [46].

### 2.2. Differential Scanning Calorimetry Measurements

**Figure 2:**
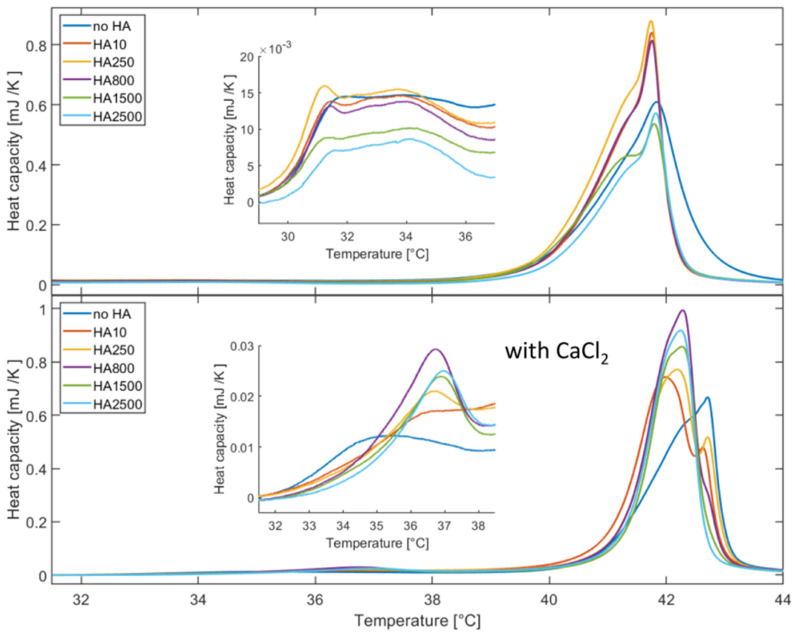
DSC measurements of DPPC vesicles with HA of varying molecular weights. (**Top**): 150 mM sodium chloride solutions. The inset shows a magnification of the pre-transition. (**Bottom**): 150 mM sodium chloride with 10 mM calcium chloride solutions. The inset shows a magnification of the pre-transition.

The DSC measurements of DPPC vesicles with HA in sodium chloride solutions are shown Figure 2 (top). Two transitions can be seen for all samples: the pre-transition around (32−34) °C and the main transition (transition from the rippled phase (P_β0_) to the liquid phase (L_α_)) slightly below 42 °C. The main transition for DPPC shows a symmetric shape with the maximum at (41.83 ± 0.02) °C. For the samples containing HA, the DSC peak becomes more asymmetric, forming a shoulder at the low temperature side. Further, a slight shift of the main transition to lower temperatures can be seen for HA with an MW lower than 800 kDa. These observations hint at a changed dynamic of the phospholipids. The pre-transition at 32 °C (see inset in Figure 2 (top)) is not very pronounced but shows two maxima. The maxima for DPPC are at (31.7 ± 0.1) °C and (34.1 ± 0.5) °C, respectively. In the presence of HA, the position of the first peak changes to values between (31.1 ± 0.1) °C and (31.5 ± 0.1) °C and the second maximum can be found between (33.8 ± 0.5) °C and (34.1 ± 0.5) °C. From the area under the complete DSC curve the enthalpy of the phase transition of DPPC (L_β0_ to L_α_) was calculated. Figure S1 (left) in the Electronic Supporting Information (ESI) shows the calculated enthalpy which for sole DPPC is (1.17 ± 0.01) mJ. Adding HA leads to an increase in the enthalpy for HA with an MW smaller than 800kDa, and has roughly the same value for HA with an MW higher than 800 kDa.

Measurements were also performed with the addition of 10 mM CaCl_2_. The DSC profiles are shown in Figure 2 (bottom) and the inset shows a magnification of the pre-transition region. The data show the same basic features (main and pre-transition peaks) as without CaCl_2_. However, differences can be observed. The temperature of the main transition of DPPC increases by 1 °C to (42.72 ± 0.02) °C compared to the solution without calcium chloride, and the peak still exhibits a symmetric shape. Adding HA leads to a decrease in the transition temperature, as seen in Figure 2. Most strikingly, a second peak develops for HA with MW lower than 800 kDa. HA with 10 kDa has the strongest effect and decreases the transition temperature by 0.7 °C. HA with MW higher than 800 kDa has similar effects and shows a decrease to a transition temperature of (42.29 ± 0.02) °C. The temperature at which the pre-transition of DPPC occurs is increased in the presence of HA (see Figure 2 (bottom inset)). Sole DPPC had a pre-transition temperature of (35.3 ± 0.1) °C, whereas the addition of HA leads to an increase in the temperature by 1.2 K for HA with 10 kDa and by 1.7 K for HA with 2500 kDa. Figure S1 (right) in the ESI shows the enthalpy of the transition of the phospholipids from the P_β0_ phase to the L_α_ phase as a function of the molecular weight. A small decrease in the enthalpy can be seen as a function of the MW of HA. The reason might be a stronger binding of HA to the DPPC head groups which constrains their mobility. We speculate that this increases the energy needed for the phospholipids to make a phase transition either from the gel phase to the rippled phase or from the ripped to the fluid phase. As the transition from the gel to rippled phase is connected with slight structural changes the effect is not as strong as for the main transition.

### 2.3. Dynamic Light Scattering

**Figure 3:**
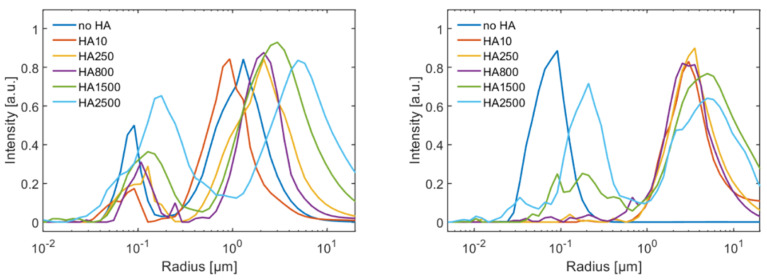
DLS data of the vesicle-HA aggregates as a function of the MW of HA. (**Left**) sodium chloride solutions (150 mM) and (**right**) sodium chloride with calcium chloride (150 mM/10 mM).

DLS measurements were performed at 25 °C. The intensity vs. radius curves are shown in Figure 3.

**Figure 4:**
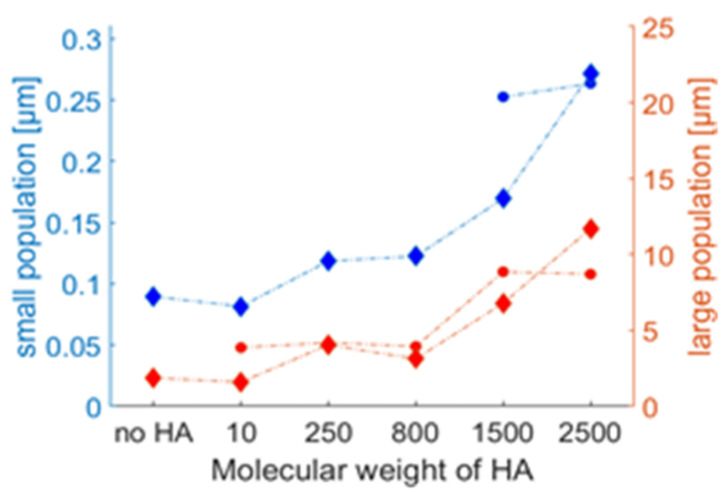
Mean size of the size populations of DPPC vesicles with and without HA as measured by DLS. Diamonds: 150 mM NaCl solutions. Circles: 150 mM NaCl and 10 mM CaCl_2_ solutions. We note that the small size population for NaCl and NaCl/CaCl_2_ coincide for no HA and the two points lie on top of each other.

Depending on the solution conditions, either a single or a bimodal size distribution can be seen. Figure 4 shows the centres of mass of the two size populations as a function of the MW of HA. The population with a radius of around 100 nm represents single vesicles, since the vesicles were formed by extrusion through membranes with a pore radius of 100 nm. A second population with micrometre size is most likely caused by aggregation of vesicles. It can be seen that the size of both populations increases with the MW of HA. The addition of CaCl_2_ modifies the size distributions, Figure 3 (right) and circles in Figure 4. Interestingly, the addition of CaCl_2_ led to the disappearance of the larger aggregates of DPPC vesicles for the solution without HA and only a population at 100 nm is present, which reflects the increased surface charge of the vesicles that counteract aggregation. Introduction of HA increases the observable size populations and aggregates reoccur. A striking difference can be observed for the samples having an MW lower than 800 kDa to the samples having a higher MW. HA with low molecular weight (≤800 kDa) in combination with CaCl_2_ causes a vanishing of the small size population at around 100 nm as can be seen in Figure 4 (right). For samples containing HA with molecular weight larger than 1500 kDa, the small size population reoccurs. For the samples with MW larger than 800 kDa, the small size population shows an increase in size from 100 nm to 250 nm. Further, the size of the larger population increases continuously with increasing MW of HA.

### 2.4. Small Angle X-ray Scattering

**Figure 5:**
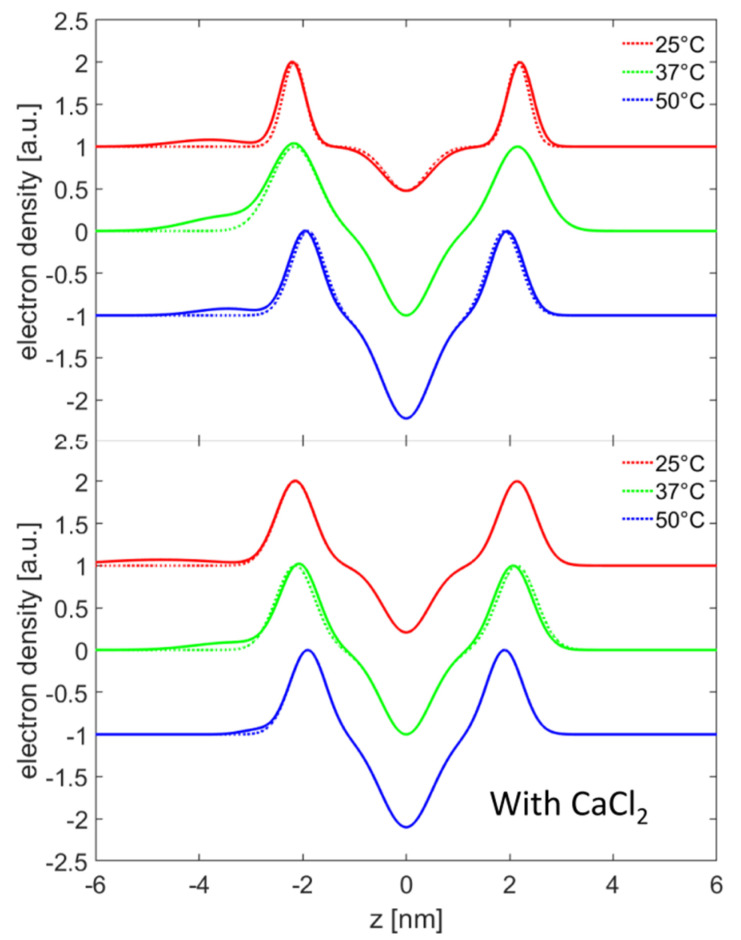
Electron density profiles obtained by the model fitting of the SAXS data on vesicles of DPPC with HA with an MW of 250 kDa (dashed lines) and without HA (solid lines). The curves were measured at different temperatures corresponding to the L_β0_ (25 °C), P_β0_ (37 °C) and L_α_ (50 °C) phase. (**Top**): 150 mM sodium chloride solutions. (**Bottom**): 150 mM sodium chloride with 10 mM calcium chloride solutions.

Structural investigations regarding the phospholipid-HA arrangement were carried out via SAXS. HA with an MW of 250 kDa was used and the measurements were performed at three different temperatures corresponding to the L_β0_ gel phase (25 °C), P_β0_ rippled phase (37 °C) and L_α_ fluid phase (50 °C). Figure S3 in the ESI shows the scattering curves acquired for each sample. The DPPC vesicles either in 150 mM sodium chloride or 150 mM sodium chloride with 10 mM calcium chloride are dominated by a broad oscillation from (0.5 to 2) nm^−1^. However, on top of the broad oscillation a small peak can be seen, indicating the existance of a fraction of multilamellar vesicles. For the analyses, the model described in the material and methods section was used. The fitting parameters are collected in Table S1, ESI; and the obtained electron density profiles are shown in Figure 5 (top dashed lines) for solution with 150 mM sodium chloride and no calcium chloride. By changing the temperature from 25 to 39 °C, a small increase in the bilayer thickness for pure DPPC can be seen, whereas the head-to-head distance z_H_ stays constant. The d-spacing increases slightly from 6.7 nm to 6.9 nm. As the temperature is increased to 50 °C, a strong decrease in the head-to-head distance (0.5 nm) as well as the d-spacing (0.6 nm) can be observed. However, at 50 °C a tendency for the formation of multilamellar vesicles is indicated by the fitting results. The ratio of unilamellar to multilamellar vesicles was calculated to be 3.6:1 at 50 °C and above 10:1 for lower temperatures.

In Figure 5 (bottom), the dashed lines represent the resulting model electron densities for the samples with CaCl_2_, and the scattering curves are shown in the ESI. While the DPPC vesicles at 25 °C and 37 °C were fitted with an exclusively unilamellar model, the model for the vesicles at 50 °C contained also a multilamellar fraction. The fits show a head-to-head distance of 4.3 nm for the bilayers at 25 °C and at 37 °C. However, the bilayer appears to have a larger thickness at 37 °C than that at 25 °C (see Figure 5 (bottom)), which is mainly caused by an increase in the width of the head group region (see parameter σ_H_ in Table S1, ESI). Further, the tail group region showed strong structural differences between 25 °C and 37 °C with a total increase in the roughness. A further increase in the temperature to 50 °C led to a clear change of the vesicle structure. The head-to-head distance decreased to 3.8 nm, a certain fraction of multillamellar structures developed with a d-spacing of 6 nm and the value of ρ_r_ increased further.

The scattering curves of DPPC with HA show a superposition of the scattering from vesicles and free unbound HA. In order to obtain only the scattering contribution from the vesicles, the scattering form HA was subtracted, which is valid for an incoherent superposition of scattering signals. The resulting scattering curves are depicted in Figures S3 and S4 in the ESI along with the scattering curves of sole HA. Since the curves for HA were almost identical it can be supposed that the structure of free HA does not change to a measurable degree as a function of temperature. The DLS measurements already indicated that HA adsorbed to the outside of the vesicles. To account for this, the model used for fitting the data was modified as described in the Materials and Methods section. The fits of the scattering curves are shown in Figures S3 and S4, ESI, and the fitting parameters are summarized in Table S1 bottom. The relative electron density profiles, shown in Figure 5 (top) by solid lines, show the presence of an additional layer on the left (outer) side of the bilayer. We note that the shape of the additional layer in the model is restricted to a Gaussian curve. Upon temperature change, it can be observed that the layer representing HA becomes more compact as the centre moves closer to the bilayer, while the thickness of the layer decreased and the electron density reached its maximum at 37 °C. In addition, the DPPC changes its bilayer thickness and the d-spacing. The d-spacing of the vesicles with HA at 37 °C (7.1 ± 0.3 nm) and 50 °C (6.6 ± 0.2 nm) was slightly increased compared to DPPC vesicles without HA (6.9 ± 0.2 nm at 37 °C and 6.3 ± 0.2 nm at 50 °C). At the same time the head-to-head distance of the bilayer with HA at 37 °C is slightly smaller than the head-to-head distance of a bilayer without HA. The structural differences between sole DPPC vesicles and DPPC vesicles with HA are quite small and special care has to be taken when interpreting the electron density profiles. Samples that also contain 10 mM CaCl_2_ in the solution show a changed slope of the scattering curves as peaks stemming from multilamellar structures disappear (see Figure S3 ESI). The width of the oscillation increases and is also better defined, indicating a lower number of lamellae and an increased ratio of unilamellar to multilamellar vesicles, respectively. The data along with the fits are shown in the ESI, Figure S3. The fitting parameters are collected in Table S2 in the ESI bottom. No major change of the vesicle structure is observed due to the presence of HA, but a significant transition from a unilamellar to a multilamellar system can be detected. Most interestingly, the data can be modelled only by a combination of unilamellar and bilamellar vesicles. The ratio between the uni- to bilamellar vesicles changes from 3:1 (25 °C and 37 °C) to 5:1 (50 °C). The structural parameters, such as the head-to-head distance, are comparable to vesicles of DPPC without the presence of HA being 4.4 nm at 25 °C, 4.3 at 37 °C and 3.9 nm at 50 °C. On the other hand, the d-spacing is increased in the presence of HA.

### 2.5. Small Angle Neutron Scattering

**Figure 6:**
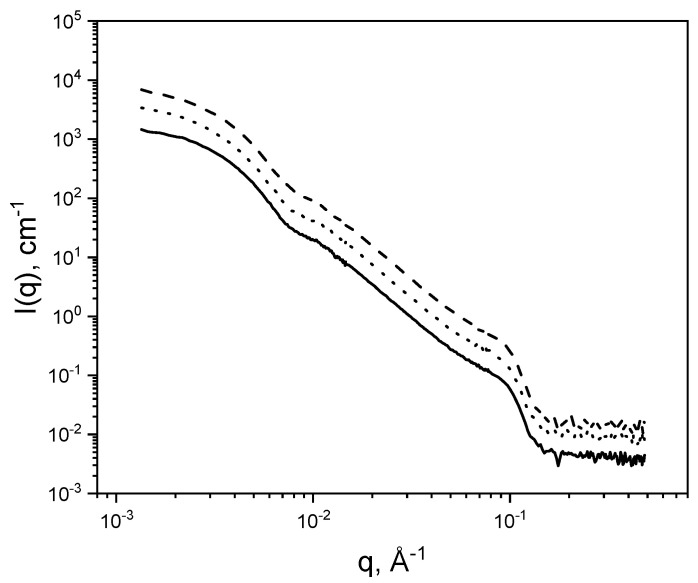
SANS scattering curves of DPPC (solid line), DPPC and HA with MW of 1500 kDa (dashed line) and DPPC with HA with MW of 10 kDa (dotted line) in 150 mM NaCl/D_2_O. Scattering data of DPPC with HA of 1500 kDa and DPPC with HA of 10 kDa have been shifted vertically (multiplied by 2 and 4, respectively) for better comparison.

The interaction of DPPC with HA with an MW of 10 kDa and HA with an MW of 1500 kDa in 150 mM NaCl/D_2_O at 50 °C was investigated by small angle neutron scattering. Due to a very weak neutron scattering contrast for HA, the scattering from DPPC is dominating and no direct contributions from HA can be observed (Figure 6). Addition of HA does not change the scattering intensity for q > 0.004 Å^−1^. This suggests that both systems (with and without HA) have the same structural features for a length scale smaller than 80 nm. From this, we conclude that the mean thickness and the curvature of the vesicles do not change as HA is added and the structure of the phospholipid layers is not changed. A difference is observed only at the lowest q-values, which corresponds to the interaction among vesicles. The determination of the apparent radius of gyration suggests an increase from (56 ± 1) nm (DPPC alone) to (60 ± 1) nm (DPPC with a HA with MW of 1500 kDa) and (61 ± 1) nm (DPPC with HA with MW of 10 kDa). This observation suggests that the structure of the phospholipid layer is constant as HA is added. We would like to point out that this is not in contradiction to the SAXS data as there is also no difference between the DPPC phospholipid bilayer structure in the presence and absence of HA was observed. Due to the low neutron scattering contrast of HA, the formed HA layer at the vesicle surface could not be detected.

### 2.6. Molecular Dynamics Simulations

**Figure 7:**
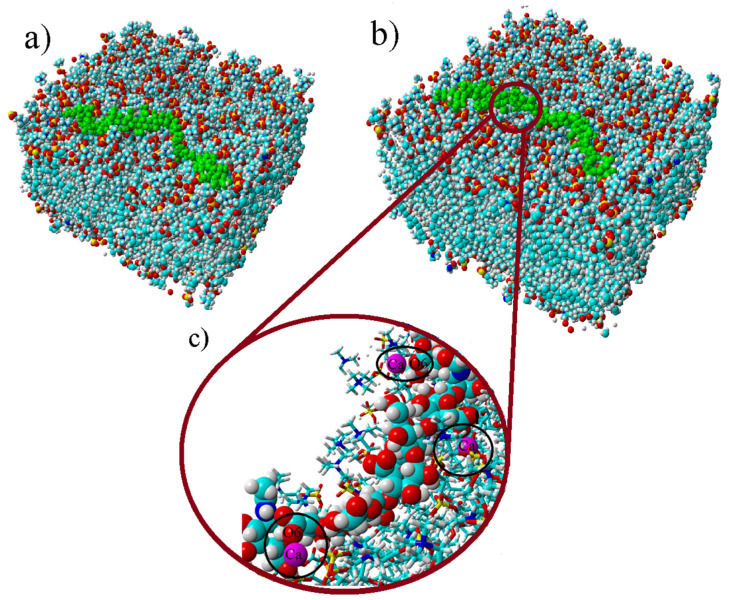
Snapshot from the simulation box: (**a**) initial position, (**b**) final structure. Green polymer represents HA. DPPC lipids are colored in the following way: hydrogen (white), carbon (turquoise), oxygen (red), nitrogen (blue) and phosphorus (yellow). (**c**) Enlarged snapshot from the simulation box at the final state. Phospholipids are represented in line connected structure, HA and Ca^2+^ as beads. In the picture we see simulation results in the presence of CaCl_2_ at 37 °C, and some calcium ions are found within the black circles in panel C.

**Figure 8:**
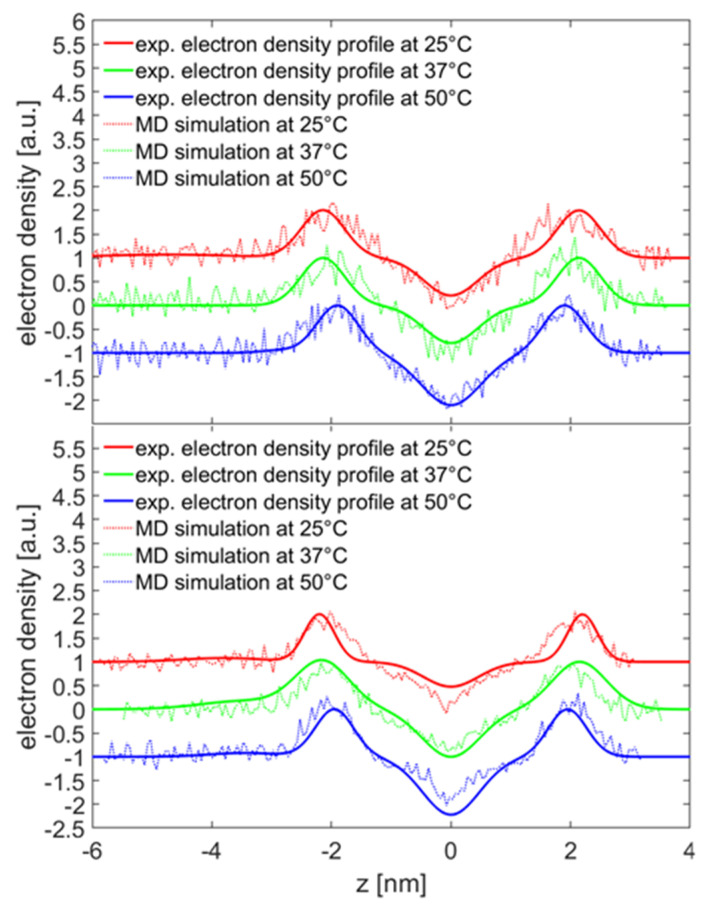
Electron density profile of the simulated DPPC bilayer with adsorbed HA.

MD simulations were carried out in order to gain understanding of how HA binds to DPPC bilayers. For this purpose, a HA with low MW (3 kDa) was utilized. Snapshots of the simulation box of the initial and final state in CaCl_2_ solution are shown in Figure 7. HA was adsorbed at the membrane surface throughout the simulation. We also calculated the electron density profiles on the basis of the simulations for the phospholipid bilayer with the adsorbed HA. Figure 8 shows the extracted profiles along with the electron density profiles obtained by the SAXS measurements. The datasets show a good agreement, validating that the simulation resembles the structure of DPPC–HA vesicles in solution.

**Figure 9:**
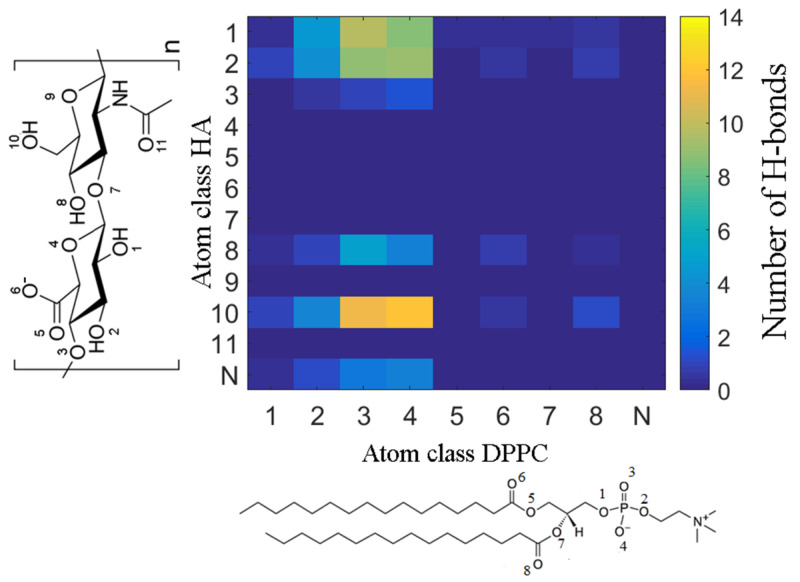
Map showing the number of HA-phospholipid H-bonds between different atom classes in HA. In the graph, HA (*y*-axis) and DPPC (*x*-axis) in NaCl solution at 37 °C are shown. The color scale indicates the number of H-bonds.

The number of HA-phospholipid-hydrogen bonds for the different atomic species was extracted and the data are plotted in Figure 9. We note that the same calculations were performed for solutions with calcium chloride and at different temperatures (25 °C, 37 °C, 50 °C). Although the simulations performed in the presence of calcium show a larger number of formed H-bond and hydrophobic contacts, no major difference could be seen for the H-bond distribution between HA and DPPC.

**Figure 10:**
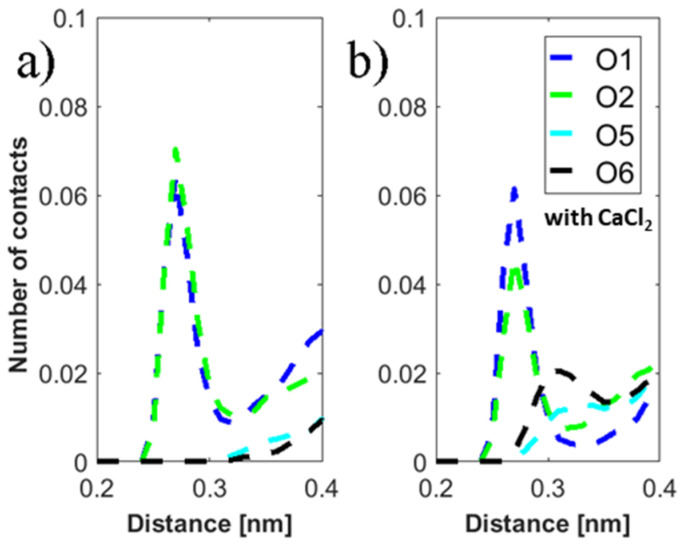
Distribution of distances between the DPPC O4 atom in the phosphate moiety and HA atoms in aqueous: (**a**) NaCl, (**b**) CaCl_2_ solutions at 37 °C. This oxygen class of DPPC was chosen since it frequently participates in H-bonds with the classes of atoms in HA as shown in Figure 8. Additionally, carboxyl group atoms (O5 and O6) are presented as calcium ions cause a shift toward the DPPC bilayer.

Figure 10 shows the extracted radial distribution functions around the DPPC O4 atom in the phosphate group and the important oxygen groups in HA, evaluated in sodium chloride and calcium chloride solutions. Along with the previous studies, we have chosen DPPC O4 as this group is frequently participating in H-bonds with HA [44] (for the numbering of the single oxygen species see Figure 9). Most of the hydrogen bonds between HA and DPPC at higher temperatures are mediated via the O10 oxygen in HA. In solutions containing calcium ions the same situation can be observed. However, the HA O5/O6 group is moved close to DPPC O4, indicating stronger association between DPPC and HA, as also suggested by the experiments.

**Figure 11:**
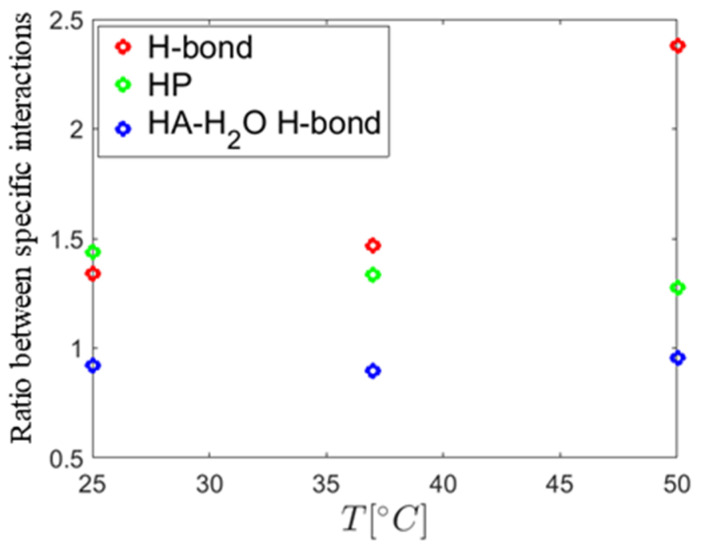
Ratios between the number of specific interactions found in CaCl_2_ and NaCl solutions as a function of temperature. The hydrogen bonds between DPPC and HA are denoted by H-bond, the hydrophobic contacts between HA and DPPC are denoted by HP. The hydrogen bonds between water and HA are denoted by HA-H_2_O.

Figure 11 reports on the ratio of the number of (i) H-bonds between HA and DPPC, (ii) hydrophobic contacts between HA and DPPC, and (iii) hydrogen bonds between water and HA molecules in calcium and sodium containing solutions. The data show that the presence of calcium ions increases the number of hydrogen bonds formed between HA and DPPC by 30% (25 °C) to 50% (37 °C). For 50 °C, this increase is as high as 150% which indicates that calcium ions may play a significant role in the interaction at high temperatures. The number of hydrophobic contacts also increased at room temperature in the presence of calcium ions, but such contacts decrease in number with temperature. We speculate that the closer contact between HA and DPPC is the reason for the lower number of hydrogen bonds between HA and water in calcium chloride than in sodium chloride solutions.

**Figure 12:**
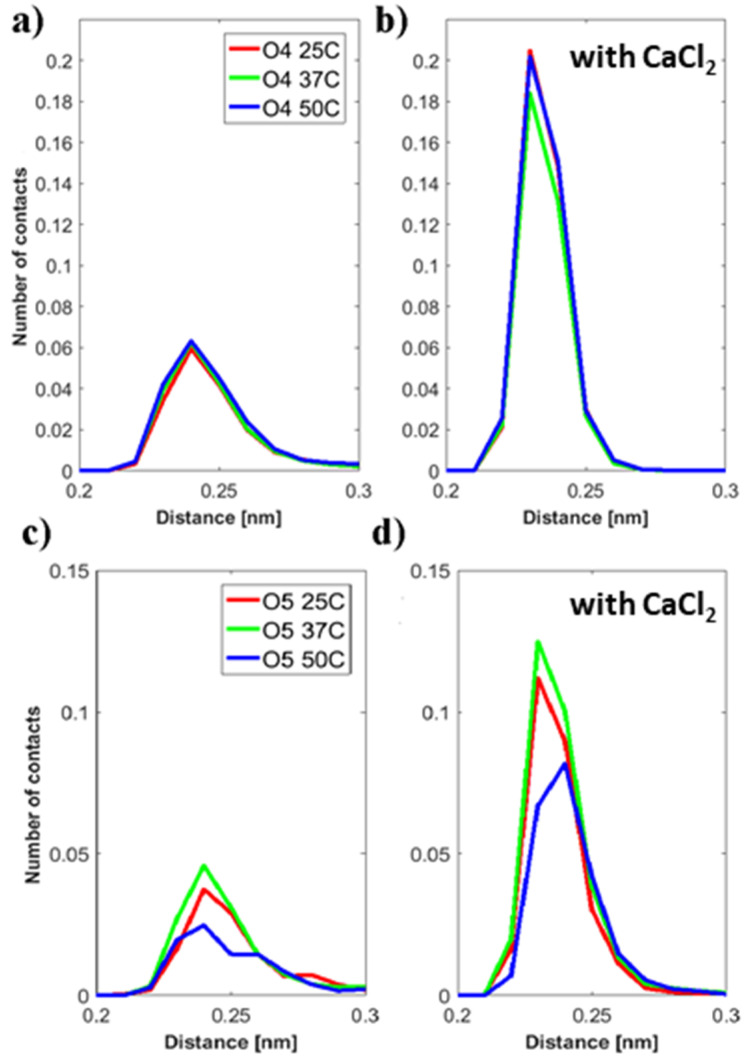
Distribution of distances between DPPC-O4 atom class and: (**a**) sodium ions, (**b**) calcium ions. Distribution of distances between: (**c**) sodium ions, (**d**) calcium ions and the HA-O5 atom class.

Figure 12a,b show the distribution of the distances between the sodium and calcium ions to the O4 oxygen of the DPPC phosphate group. It can be seen that significantly more calcium ions are found close to the O4 oxygen of the phosphate group. Calcium ions are also found slightly closer to the oxygen group as the peak is observed at ~2.3 Å, whereas for sodium it is at ~2.4 Å.

We also considered the distance distribution of sodium and calcium ions relative to the carboxylate oxygen O5 of HA, see Figure 12c,d. Two observations can be made: First, there are significantly more calcium ions close to the O5 carboxylate oxygen than sodium ions. Second, the number of ions close to the carboxylate O5 of HA is decreasing with temperature, and the temperature-dependence is stronger for calcium ions than for sodium ions. The reduction in the number of contacts observed at 25/37 °C and at 50 °C is a result of a less tight association between HA and the DPPC bilayer in the calcium containing bilayer. This indicates a more preferred binding of calcium ions to DPPC than to HA, which is consistent with the electrophoretic mobility data.

## 3. Discussion

The DSC measurements show two characteristic peaks, one for the pre-transition and one for the main transition which is in agreement with the literature [49,50]. However, the data indicate that an interaction of HA with the glycerol backbone at the hydrophobic–hydrophilic interface, as a shoulder, is present at the main transition [51]. This is a realistic scenario for HA as it has been reported that HA has hydrophobic patches, which can bind to phospholipids [20,52]. However, in the simulation results reported here, hydrophobic interactions between HA and DPPC are identified exclusively with the CH_3_-groups present in the head group. An interaction of HA with the alkyl chains of the lipids would induce a change of the main transition temperature and, in parallel, an increase in the width of the main transition [51]. As we only observe a slight shift of the main transition along with a change in the transition enthalpy it can be ruled out that HA interacts with the tail group of DPPC.

Further, the DSC data also show that calcium ions significantly change the interactions in the DPPC and DPPC/HA systems. Our data demonstrate that Ca^2+^ binds to the DPPC bilayer and thereby affects the phase behavior. The addition of HA leads to a strong decrease in the pre- and main-transition, which is significantly stronger for HA with an MW lower than 800 kDa. Such changes are normally regarded as a sign for impurities disturbing the alkyl chain packing [50]. However, as also the shapes of the transition peaks are changed, while in parallel a second peak at the main transition occurs; thus, it can be deduced that HA only interacts with the outer headgroup [51] as detailed in the simulation work reported here. We speculate that this interaction changes the mobility and degree of freedom of the DPPC molecules in the outer layer, making the system more stiff and inflexible. The appearance of the second peak can be understood in terms of two phases being present: one in the inner DPPC layer of the vesicles not interacting with HA and one associated with the outer layer affected by HA adsorption. This leads to a different phase behavior and a decoupling of the bilayer leaflets as observed in other studies [47]. On the basis of the electrophoretic mobility measurements, we postulate that the binding to the outer layer is favoured by electrostatic interactions, but MD simulations also demonstrate hydrogen bonding and hydrophobic interactions between HA and DPPC bilayers. In solutions containing both HA and DPPC, the charge of the aggregates is less than that of the vesicles in absence of HA, indicating partial charge compensation only possibly by the binding of HA to DPPC.

The DLS measurements show that addition of HA, both in presence and absence of calcium ions, leads to the increase in the hydrodynamic radius of the vesicles. Further vesicle aggregation occurs, and the extent of aggregation depends on the MW of HA. As discussed above, the DSC also indicates a change in the interaction depending on the MW. The transition enthalpy decreases for HA with an MW higher than 800 kDa, which indicates a modified interaction mechanisms. This can be understood by the change of the polymer chain conformation as high and low MW of HA have the same structure and number of charged carboxylate groups per monomer. At low MW, HA can be regarded as a short chain having no or few intramolecular interactions. This changes as the chain gets longer and becomes progressively more able to coil and develop intramolecular interactions that reduce the ability of HA to interact with the DPPC bilayer. Further, different hydrodynamic radii of the vesicles as a function of the MW of HA can be understood by the different conformations of the HA polymer chain. HA with an increased MW will lead to a larger increase in the hydrodynamic radius due to the larger extension of the polymer chain into the solution. Our data also suggest that a critical MW for this change in the interaction is around 800 kDa, but it is as yet not clear why this transition occurs at such a high molecular weight.

The SAXS measurements on DPPC vesicles with HA were performed at three bilayer phases: gel phase (L_β0_), rippled phase (P_β0_) and fluid phase (L_α_) [53]. The different phases have different structural conformations, and the most clearly observed change was a reduction in the d-spacing and head-to-head distance as the bilayer makes a transition to the L_α_ phase. The obtained values for the head-to-head distance in the different phases agree well with the literature data [54]. However, the results for the d-spacing do not match. A possible reason might be the preparation method as in this study vesicles with a diameter of 100 nm were used. Such vesicles could have high stresses due to the high curvature which is expected to have an impact on the d-spacing [55]. The fits show that the electron density ρ_T_ of the phospholipids increase as the L_α_ phase is reached which can be due to a changed packing of the DPPC molecules in the vesicles due to a higher disorder in the bilayer as a result of the melting of the alkyl chains. Such an increased volume per molecule is also reported in the literature [56]. Upon the addition of HA, no detectable change of the vesicle bilayer structure can be observed as the scattering curves are similar to each other and, thus, the obtained electron density profiles are indistinguishable. A diffuse layer of HA can, however, be seen, which is most compact at 37 °C where the DPPC vesicles are in the rippled phase. However, in the presence of HA an increased d-spacing can be recognized for the P_β0_ and the L_α_ phases. This hints at an accumulation of HA in between the bilayer as reported by Kreuzer at al. [23]. It remains an open question how HA enters the inter bilayer region, as vesicles consist of closed lipid shells and an HA-induced disruption of the bilayer seems unlikely. A scenario where HA penetrates thought the membranes can be ruled out by the DSC measurements. Some parts of the multillamellar structure could arise from aggregates of vesicles where it is possible for the HA to accumulate between bilayers.

In the presence of 10 mM, only CaCl_2_ unilamellar vesicles can be found as also reported in the literature [39]. In contrast to the cited work, oligo lamellar structures could be detected at 50 °C, which could be due to the high concentration of NaCl used in our study [47]. In addition to promoting the formation of unilamellar vesicles, the addition of Ca^2+^ also has a strong effect on the electron density in the tail group region. The higher electron density level indicates a higher packing of the lipids induced by Ca^2+^, as already suggested by Kataoka et al. 1985 [34] and Aruga et al. 1985 [33]. The extracted head-to-head distance in the three different phases for bilayers in contact with 150 mM NaCl solutions, in absence and presence of 10 mM CaCl_2_, showed no effect due to calcium ions. This is only partially consistent with the reports in the literature [39]. Here, an induced increase in the bilayer thickness in the L_α_ phase of about 0.2 nm was observed in the presence of 10 mM CaCl_2_. The origin was speculated to be a changed packing induced by the electrostatic potential of the adsorbed Ca^2+^ ions. However, other studies do not report any change due to the presence of Ca^2+^ [36].

When both calcium ions and HA are present, a layer of HA could be detected which had a more compact structure than in sodium chloride solutions, indicating a stronger interaction between the DPPC bilayer and HA chains, which is also observed in the MD simulations. The population of pure unilamellar vesicles changed and bilayer structures could be detected. The question arises if the presence of the double bilayer vesicles is a consequence of disruption and reorganization of vesicles due to HA or if HA induced an aggregation with a favorable distance between bilayers. The aggregation could also be seen by DLS. However, for a disruption of the vesicles, a strong interaction of HA and the alkyl chains has to be present, which can be ruled out by the DSC and small angle scattering measurements.

The MD simulations yield insight to the direct binding of DPPC and HA. As one can see in Figure 8, the conformation of HA was not significantly changed during the simulation at 37 °C in calcium chloride solution. However, for other cases, HA was more detached from the surface. In general, no strong effect on the binding distribution behavior could be observed for the simulated conditions, which can be a result of the short chain used. However, for conditions where calcium is present, a total increase in the number of interaction points, either by hydrophobic contacts or HA–DPPC hydrogen bonds, is observed, which can be interpreted as adsorption of HA closer to the bilayer, which is consistent with the enhanced adsorption observed by the experiments. At 50 °C, the relative number of H-bonds formed in calcium ion containing solution is higher than in sodium ion containing solutions, indicating a stronger impact of calcium ions on the binding behavior at higher temperatures. In the presence of calcium ions, the number of hydrogen bonds to water for HA is decreased. This can be explained by the binding of calcium ions to HA and the closer binding of HA to the DPPC bilayer in the presence of calcium ions, which reduce the possibility for water binding [44]. The distribution of distances of ions to the HA-O5 (carboxylate) and DPPC-O4 (phosphate) atoms shows more calcium ions than sodium ions in close proximity to these groups. Further, calcium-ion-mediated ionic bridges between these groups were observed. Such bridges may stabilize the DPPC–HA binding and thereby lead to an increased number of H-bonds and hydrophobic contacts. This property might be of higher importance at elevated temperatures as indicated by the increase in the relative number of hydrogen bonds between DPPC and HA at 50 °C and might explain the change in the DSC data. For HA, a similar behavior is seen as the calcium ions are also moved closer to the carboxyl group due to electrostatic attraction. This can also explain a lower binding of water molecules to the HA-O5 atom as calcium ions are more directly involved in electrostatic interaction and, thus, result in a shift of water away from carboxyl group.

## 4. Materials and Methods

### 4.1. Materials

DPPC was purchased from Avanti polar lipids (catalogue No. 850355P) (Alabaster, AL, USA), and HA was bought from Creative PEGworks (Durham, NC, USA). HA samples with weight averaged molecular weights of 10 kDa (Catalogue No. HA-101), 250 kDa (catalogue No. HA-103), 800 kDa (catalogue No. HA-104), 1500 kDa (catalogue No. HA-106) and 2500 kDa (catalogue No. HA-107) were used. All compounds were used as received. In the following, HA with an MW of 10 kDa will be referred to as HA10, HA with an MW of 1500 kDa as HA1500 and so on. Sodium chloride (assay ≥99.8, catalogue No. 31434, purchased from Sigma-Aldrich, Munich, Germany), calcium chloride dehydrate (assay ≥99, catalogue No. 5239, purchased from Carl Roth, Roth, Germany) and ultrapure water (Milli-Q, resistivity 18.2 MΩ cm) were used to prepare all aqueous solutions. The electrophoretic mobility measurements were performed with sodium hyaluronate (HA) with a molecular weight of about 0.9 × 10^6^ Da, which was received as a gift from Novozymes (Nottingham, UK).

### 4.2. Sample Preparation

The desired amount of lipid powder was dissolved in chloroform and transferred into a glass vial. The chloroform was then evaporated under a flow of nitrogen, whereby a lipid film formed on the walls of the glass vial. Residual chloroform was removed by keeping the vials in an oven over night at 60 °C. Next, the aqueous solution was added, either 150 mM NaCl or 150 mM NaCl with 10 mM CaCl_2_, to achieve a target concentration of the phospholipids of 10 mg/mL. The solution was heated to 55 °C and vortexed for 5 min, which resulted in a turbid solution of multillamellar vesicles with broad size distribution. To achieve further dispersion of the phospholipid aggregates, the samples were kept at 55 °C in a thermomixer (BioShake Series, Analytik Jena, Jena, Germany) using a rotation rate of 350 rpm for 3 h, interrupted by vortexing each hour. In the final step, the multillamellar vesicles were extruded 35 times through a membrane with 0.2 µm pore size (Nuclepore Track-Etched Polycarbonate Hydrophilic Membranes, GE Healthcare Life Science, Little Chalfont, UK) making use of the Avanti Mini-Extruder (Avanti Polar Lipids Inc., Alabaster, AL, USA) in order to form almost unilamellar vesicles. Extrusion was carried out at 55 °C and the solution was kept at 55 °C until use to prevent any aggregation. The DSC data that are consistent with the literature data suggest that hydrolysis of DPPC at the high temperature is negligible.

For DPPC-vesicle/HA samples, a HA solution was prepared by adding HA to the aqueous salt solution, and the solution was stirred until HA was completely dissolved. The mass concentration of HA in the solution was set to be equal to the mass concentration of DPPC, both being 8 mg/mL. Finally, the HA solution was mixed with the vesicles solution in a ratio of 1:1 at 55 °C and kept at this temperature until use.

### 4.3. Small Angle X-ray Scattering and Analysis

The small angle X-ray scattering experiments were performed at the P12 beamline, Petra III, DESY, Hamburg, Germany, using an X-ray energy of 10 keV [57]. The samples and the aqueous salt solution for background correction were measured in glass capillaries with a diameter of 1 mm and wall thickness of 0.01 mm. A Linkam (Tadworth, UK) heating stage was used for temperature control. Measurements were performed at: 25 °C, 37 °C and 50 °C. DPPC and DPPC/HA samples were studied in solutions conditions of 150 mM in absence and presence of 10 mM CaCl_2_. The concentration of DPPC and HA was 4 mg/mL, respectively, and HAs with different molecular weights were used.

To fit the SAXS data, a model electron density profile was built and a scattering intensity curve was calculated. This curve was fitted to the measured datasets of the samples. The scattering curves of the DPPC-vesicles usually show two characteristic kinks at q ≈ 1 nm^−1^ and q ≈ 1.81 nm^−1^, which are clear signs for the existence of “multi” lamellar structures. Therefore, a combination of form factors was used to account for the simultaneous presence of “multi” and single bilayer structures. Different electron density profiles were used for the form factors. We used a model developed by Pabst et al. [58]. Here, a single bilayer structure is described by a combination of three Gaussian curves with two curves for the heads and one curve for the tails:

(1) $$\rho_{S}\left(z\right){=\rho }_{\mathrm{Water}}+\;∆\rho_{H}e^{-\frac{{\left({z-z}_{H}\right)}^{2}}{{2\sigma }_{H}}}+∆\rho_{T}e^{-\frac{{\left(z\right)}^{2}}{{2\sigma }_{T}}}+∆\rho_{H}e^{-\frac{{{(z+z}_{H})}^{2}}{{2\sigma }_{H}}}$$

The electron density profile is symmetric with its centre set to z = 0. The distance between the head group and the centre is denoted by z_H_. The parameters σ_H_ and σ_T_ describe the width of the Gaussian curves for the head group and tail group, respectively. Δρ_H_ and Δρ_T_ are defined as the difference between the electron density of the head groups and the tail groups to the electron density of water Δρ_H/T_ = ρ_H/T_ − ρ_Water_. For a further reduction in the parameter space, ρ_s_(z) is normalized by Δρ_H_, which leaves only one parameter Δρ_r_ describing the ratio of the electron densities of the head groups and tails:

(2) $$\rho_{r}=\frac{\left|\rho_{\mathrm{tails}}{-\;\rho }_{\mathrm{water}}\right|}{\left|\rho_{\mathrm{heads}}{-\rho }_{\mathrm{water}}\right|}$$

Thus, ρ_s_(z) can now be written as [58]:

(3) $$\rho_{S}\left(z\right){=\;e}^{-\frac{{\left({z-z}_{H}\right)}^{2}}{{2\sigma }_{H}}}+∆\rho_{r}e^{-\frac{{\left(z\right)}^{2}}{{2\sigma }_{T}}}{+e}^{-\frac{{{(z+z}_{H})}^{2}}{{2\sigma }_{H}}}$$

In order to account for a double bilayer or multilayer structure, *ρ*_d_, the structure of the single bilayer, was doubled, and an additional parameter *d* was introduced, which describes the distance between the centres of the bilayers (repeat distance):

(4) $$\rho_{D}\left(z\right)=\;e^{-\frac{{\left({z-z}_{H}-\frac{d}{2}\right)}^{2}}{{2\sigma }_{H}}}+∆\rho_{r}e^{-\frac{{\left(z-\frac{d}{2}\right)}^{2}}{{2\sigma }_{T}}}{+e}^{-\frac{{{(z+z}_{H}-\frac{d}{2})}^{2}}{{2\sigma }_{H}}}{+e}^{-\frac{{\left({z-z}_{H}+\frac{d}{2}\right)}^{2}}{{2\sigma }_{H}}}+∆\rho_{r}e^{-\frac{{\left(z+\frac{d}{2}\right)}^{2}}{{2\sigma }_{T}}}{+e}^{-\frac{{{(z+z}_{H}+\frac{d}{2})}^{2}}{{2\sigma }_{H}}}$$

To calculate the scattered intensity of mixtures of lamellar structures, the different form factors have to be summed and the following formula is used:

(5) $$I\left(q\right)=A\;\frac{1}{q^{2}}\left({n|F}_{S}|{}^{2}+\overset{x}{\underset{a=2}{\sum }}{|F}_{a}|{}^{2}\right)+bkg$$

*A* is a scaling factor, with *bkg*, a constant, taking into account the incoherent background. The factor *n* scales the amount single bilayer and multilayer structures with a number of *x* layers. This term can be expanded to also include the desired number of multilayers of the system to be analyzed. This model has the advantage that the overall scattering intensity is calculated by using individual form factors for each structure, and no structure factor for multillamellar structures is needed. For mixtures with a maximum number of *x* layers, the ratio of unilamellar to multilamellar vesicles *R* can be calculated:

(6) $$R=\;\frac{n}{x-1}$$

The use of a combination of a second form factor for the double bilayer instead of introducing a structure factor has the advantage that it is possible to add an extra layer to account for the adsorption of HA to the outer vesicle shell. Here, adsorption can only occur to one side of the bilayer structure as HA was added after the vesicles were formed. The adsorbed layer (HA) was also described by a Gaussian function and added to the ρ_s_(z) and ρ_d_(z) profiles from Equations (3) and (4) to yield:

(7) $$\rho_{D-\mathrm{HA}}\left(z\right){=\;\rho }_{D}\left(z\right){+\rho }_{\mathrm{rHA}\;}e^{-\frac{{\left({z-z}_{\mathrm{HA}}\right)}^{2}}{{2\sigma }_{\mathrm{HA}}}}$$

(8) $$\rho_{S-\mathrm{HA}}\left(z\right){=\;\rho }_{S}\left(z\right){+\rho }_{\mathrm{rHA}\;}e^{-\frac{{\left({z-z}_{\mathrm{HA}}\right)}^{2}}{{2\sigma }_{\mathrm{HA}}}}$$

With ρ_rHA_ being defined as

(9) $$\rho_{\mathrm{rHA}}=\frac{{|\rho }_{\mathrm{HA}}{-\;\rho }_{\mathrm{water}}|}{{|\rho }_{\mathrm{Heads}}{-\rho }_{\mathrm{water}}|}$$

The fitting was performed with MATLAB (MATLAB version 8.2, MathWorks, Natick, MA, USA) making use of the non-linear curve-fitting routine “lsqcurvefit” (part of the MATLAB Optimization Toolbox), which uses a least-square method.

### 4.4. Small Angle Neutron Scattering

SANS experiments were performed at the D11 instrument of the Institute Laue Langevin (Grenoble, France) [59]. Three q-ranges were explored and merged using the following wavelengths, λ, and sample-to-detector (SD) distances. The q-ranges were (1) low-q: λ = 6 Å, SD = 39 m; (2) mid-q: λ = 6 Å, SD = 8 m; (3) high-q: λ = 6 Å, SD = 1.2 m. Solutions and backgrounds were examined in standard 1 mm quartz cells. Direct beam, empty cell, and Teflon were recorded and boron carbide (B_4_C) was used as neutron absorber. All samples were kept at 50 °C (above the phase transition temperature of the phospholipid) during the measurements. The scattering from the background sample was subtracted from the experimental data. Absolute values of the scattering intensity were obtained from the direct determination of the number of neutrons in the incident beam and the detector cell solid angle. The 2-D raw data were corrected for the ambient background and empty cell scattering and normalized to yield an absolute scale (cross section per unit volume) by the neutron flux on the samples. The data were then circularly averaged to yield the 1-D intensity distribution, I(q). Samples were prepared in 150 mM NaCl/D_2_O (99.9%) to limit the incoherent background scattering. The concentration of DPPC and HA (M_W_ = 10 kDa and M_W_ = 1500 kDa) was the same and equal to 2 mg/mL. The full set of experimental data can be found in [60].

### 4.5. Electrophoretic Experiments

Electrophoretic mobility measurements were performed at 25 °C, employing a Zetasizer 2000 instrument (Malvern Instruments, Malvern, UK). Each sample was measured 15 times, and the mean value for three independent samples is reported.

### 4.6. MD Simulations

All atom molecular dynamics simulations were performed using the AMBER03 force field [24]. The HA structure was downloaded from PubChem [26] and modified to obtain longer chains by using the YASARA (Yet Another Scientific Artificial Reality Application) Structure Software (YASARA Biosciences GmbH, Vienna, Austria). The final molecular mass of HA considered in this study was 3 kDa. The DPPC bilayer contained 288 lipid molecules. After allowing the bilayer to equilibrate, the HA molecule was placed next to the bilayer without influencing the bilayer structure. Periodic boundary conditions were applied to create an “infinite” bilayer. The TIP3P water model was used [28]. For the isobaric–isothermal ensemble, all atom simulations were performed under the same conditions: temperatures: 298, 310 (physiological) and 323 K at a pH = 7.0 in 150 mM NaCl and CaCl_2_ water solutions at a pressure of 1 bar. The time step was set to 2 fs. The simulation box contained water, one HA molecule, a DPPC bilayer, 56 cations and the corresponding number of anions to achieve electro neutrality. Simulations were carried out for 20 ns. The Berendsen barostat [29] and thermostat with a relaxation time of 1 ps were used to maintain constant pressure and temperature. Electron density profile was calculated by using the Visual Molecular Dynamics (VMD) tool Electron Density Profile [61]. The definition of hydrophobic contacts and hydrogen bonds, as stated in previous studies, was used [44,45]. Hydrogen bonds, ion-DPPC/HA contacts and HA–DPPC contacts have been extracted from the equilibrated state. For this, the data were sampled every 0.05 ns and it was checked for at which time a stable state was reached. This was typically the case between (10–20 ns).

### 4.7. Dynamic Light Scattering

Dynamic Light Scattering was used to obtain information about the size of the particles in solution. Therefore, it has been used to examine the interaction of lipid vesicles and HA by studying how the hydrodynamic size of the vesicles changes due to the presence of HA.

The measurements were performed using the SpectroSize 300 (Xtal concepts GmbH, Hamburg, Germany) equipped with a laser with a wavelength of 660 nm. The scattering angle was set to 90 °C and measurements were performed at 25 °C. All sample solutions were measured in the same quartz cuvette. Each sample was measured 10 times for 20 s. Six different samples (DPPC, DPPC/HA10, DPPC/HA250, DPPC/HA750, DPPC/HA1500, DPPC/HA2500) were studied at two different solution conditions (150 mM NaCl and 150 mM NaCl with 10 mM CaCl_2_). The concentration of DPPC and HA was 1 mg/mL, respectively.

### 4.8. Differential Scanning Calorimetry

Differential scanning calorimetry (DSC) is well-suited for studying phase transitions. It was used to investigate how the presence of HA changes the phase behavior of DPPC-bilayers. For the measurements, a VP-DSC MicroCalorimeter (MicroCal, Northhampton, MA, USA) was used. The instrument is equipped with two Tantaloy 61 cells (one for the sample and one for the reference). Each sample was scanned 5 times from 20 to 55 °C and five times in the inverse direction. The scanning rate was 20 °C/h for all measurements with a waiting time of 15 min between each scan. Care was taken to have the same volume (0.5 mL) in the sample and reference cell. All solutions were degassed prior to measurements to avoid air bubble formation.

Samples composed of DPPC vesicles and DPPC vesicles with HA of different molecular weights (10 kDa, 250 kDa, 750 kDa, 1500 kDa and 2500 kDa) were probed in two different salt solutions (150 mM NaCl and 150 mM NaCl with 10 mM CaCl_2_). The concentration of the samples was 1 mg/mL for both HA and DPPC vesicles.

## 5. Conclusions

This study elucidates the molecular interactions between HA and DPPC to shed light on the synergistic interactions and adds to the understanding of the underlying interaction principles of two of the main synovial fluid components. The typical concentration of calcium ions in the synovial fluid is about 5 mM [31,32]. The data give insight to the different interactions of hydrogen bonds, hydrophobic contacts, electrostatic interactions and the influence of the MW of HA on its behavior at a DPPC bilayer. HA with low MW shows stronger interactions as compared to HA with higher MW. This causes more aggregation and a larger shift in the phase transition temperatures of DPPC. This observation is mostly induced by the different sizes of the HA, as longer HA forms a random coil with potential interaction sites being inaccessible, whereas low MW of HA has a more open conformation exposing the carboxylate units to the phospholipids. No interaction of the HA with the alkyl chain of DPPC can be seen as this would have been detected in the DSC and small angle scattering measurements as well as with the MD simulations. This is in agreement with other studies that claimed an adsorption to the headgroup [23,29]. Especially the SAS measurements, which show no influence on the structural arrangement of the alkyl chains as HA is present. Only adsorption to the outer shell of the vesicles can be detected. However, the DSC data also hint at a low amount of HA being present at the glycerol-tail group interface. The changes in the phase behavior of DPPC can therefore be attributed solely to the interaction of HA with the headgroup of DPPC. Calcium ions increase the overall interaction strength between HA and DPPC. Our data, especially the electrophoretic mobility measurements, support the idea that this is induced by an increased positive charge of the vesicles by the calcium ions which bind to the phosphate group as also observed by Bełdowski et al. [44]. The MD simulations indicate a complex interaction between HA and DPPC. Specific H-bonds are formed between H-bond donators on HA and the phosphate group of DPPC; hydrophobic interactions also contribute along with electrostatic interactions between carboxylate groups of HA and positive headgroup charges. A key finding is the observation that calcium ions are more abundant next to the carboxylate groups of HA and by this reduce the binding of water to the carboxylate group of HA. In the presence of calcium ions, ionic bridges between carboxylate ions of HA and the phosphate group of DPPC are developing, which likely is the main reason for the stronger interactions as the analyzed binding distribution is not changed. We speculate that this stronger binding is the reason for the development of two phases as observed in the DSC data.

## Supplementary Materials

The following are available online, Figure S1 Enthalpy of the main transition calculated from the DSC measurements, Figure S2 DLS data of the vesicle-HA aggregates as function of the MW of HA. Figure S3 SAXS data of the vesicle-HA aggregates as function of the temperature. Figure S4 SAXS data of HA as function of the temperature. Table S1 Parameters of the fits of DPPC vesicles with and without HA in sodium chloride solutions. Table S2 Parameters of the fits of the DPPC vesicles and DPPC vesicles with and without HA in 150 mM NaCl with 10 mM CaCl_2_.
