# Supplementary material for: Influence of the Molecular Weight and the Presence of Calcium Ions on the Molecular Interaction of Hyaluronan and DPPC
Source: Molecules. 2020 Aug 27;25(17):3907. doi: 10.3390/molecules25173907 (PMC7504306; doi:10.3390/molecules25173907)
Supplement: Supplementary file 1 [file molecules-25-03907-s001.pdf]

molecules

Article

Influence of the molecular weight and the presence of calcium ions on the molecular Interaction of hyaluronan and DPPC

T. Zander[1]. V.M. Garamus[1], Andra Dedinaite[2,3], Per M. Claesson[2,3], P. Bełdowski[4], K. Górny[5], Z. Dendzik[5], D.C.F. Wieland[1]*, Regine Willumeit-Römer[1]

[1]Helmholtz-Zentrum Geesthacht: Centre for Materials and Costal Research, Institute of Materials Research, Max-Planck-Straße 1, 21502 Geesthacht, Germany

[2] KTH Royal Institute of Technology, School of Engineering Sciences in Chemistry, Biotechnology and Health, Department of Chemistry, Surface and Corrosion Science, Drottning Kristinas väg 51, SE-10044 Stockholm, Sweden

[3] RISE Research Institutes of Sweden, Division of Bioscience and Materials, SE-114 86 Stockholm, Sweden, SE-114 86 Stockholm, Sweden

[4] Institue of Mathematics and Physcis, UTP University of Science and Technology, al. Kaliskiego 7, 85-796 Bydgoszcz, Poland

[5] University of Silesia in Katowice, Faculty of Science and Technology, 75 Pułku Piechoty 1A, 41-500 Chorzów, Poland

* Correspondence: florian.wieland@hzg.de;

Received: date; Accepted: date; Published: date

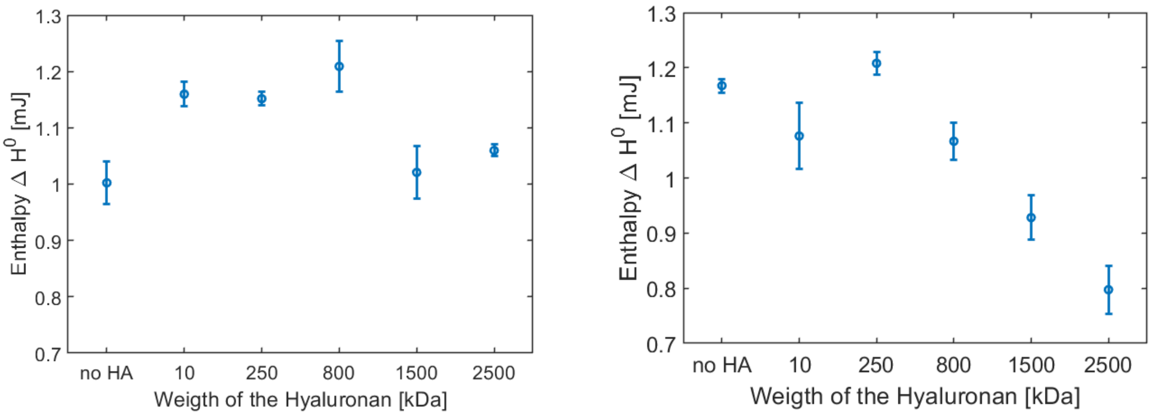

Figure S1 Enthalpy of the main transition calculated from the DSC measurements for samples in (left) sodium chloride solutions (150 mM) and (right) sodium chloride with calcium chloride (150 mM / 10 mM)

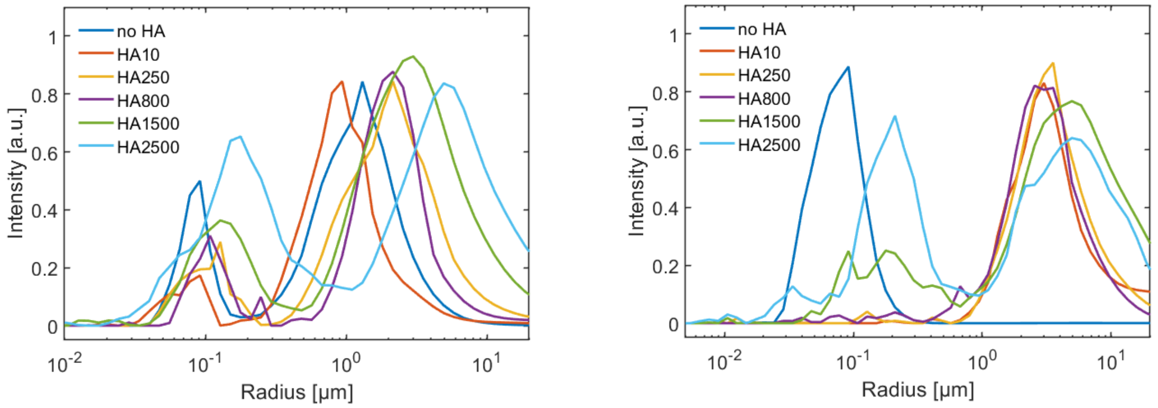

Figure S2 DLS data of the vesicle-HA aggregates as function of the MW of HA. (Left) sodium chloride solutions (150 mM) and (right) sodium chloride with calcium chloride (150 mM / 10 mM)

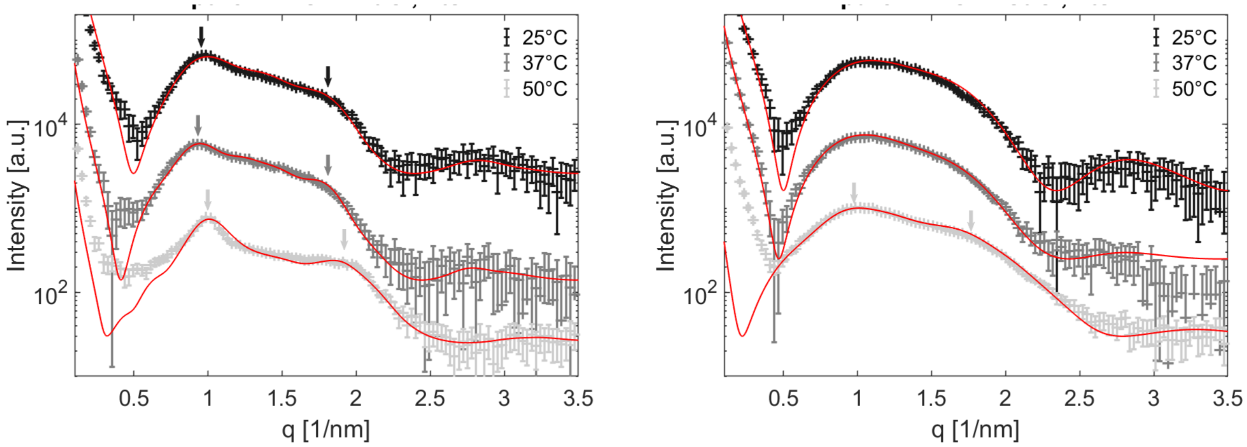

Figure S3 SAXS data of the vesicle-HA aggregates as function of the temperature. (Left) Sodium chloride solutions (150 mM) and (right) sodium chloride with calcium chloride (150 mM / 10 mM) The temperauter correspnds to the gel phase, rippled phase and fluid phase.

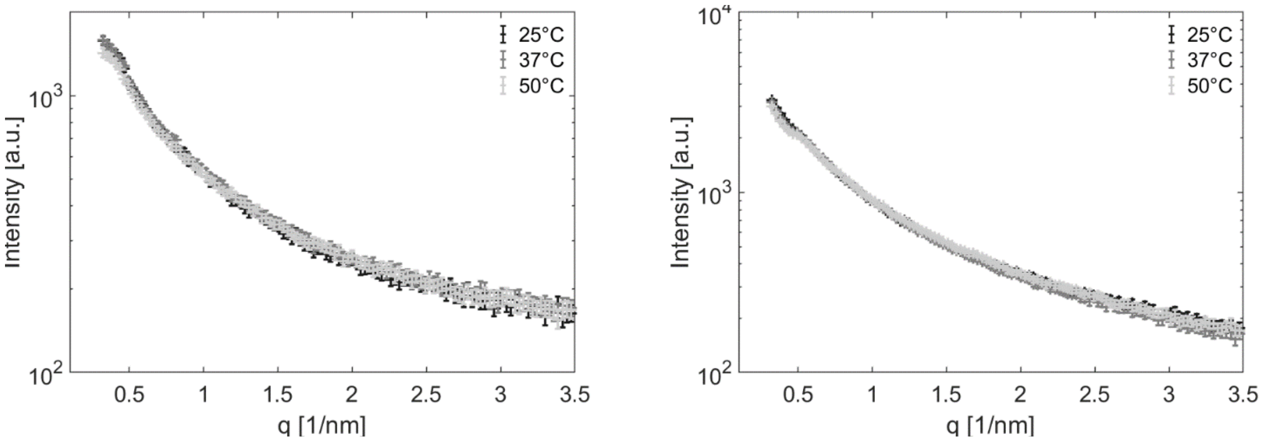

Figure S4 SAXS data of HA as function of the temperature. (Left) Sodium chloride solutions (150 mM) and (right) sodium chloride with calcium chloride (150 mM / 10 mM) The temperauter correspnds to the gel phase, rippled phase and fluid phase.

Withot HA	25°C	37°C	50°C
σ_H [nm]	0.36± 0.02	0.38± 0.02	0.35± 0.03
σ_T [nm]	0.45± 0.02	0.48± 0.03	0.50± 0.04
z_H [nm]	2.14± 0.04	2.14± 0.04	1.10± 0.07
ρ_r	0.79± 0.04	1.00± 0.06	1.10± 0.07
d [nm]	6.7 ± 0.2	6.9 ± 0.2	6.3 ± 0.2
n	20 ± 1	45 ± 2	18 ± 2
A	6.5 ± 0.5	2.1 ± 0.1	4.1 ± 0.3
BG	26 ± 3	14 ± 1	25 ± 3
No. Layers	3	4	5
With HA	25°C w HA	37°C w HA	50°C w HA
σ_H [nm]	0.36± 0.01	0.38± 0.02	0.35± 0.03
σ_T [nm]	0.45± 0.03	0.48± 0.02	0.5 ± 0.03
z_H [nm]	2.14± 0.04	2.06± 0.03	1.90± 0.05
ρ_r	0.79± 0.03	1 ± 0.04	1.10± 0.05
d [nm]	6.7 ± 0.2	7.1 ± 0.3	6.6 ± 0.2
n	19 ± 2	29 ± 2	29 ± 2
A	6.2 ± 0.3	3.1 ± 0.1	3.2 ± 0.2
BG	22 ± 3	17 ± 2	25 ± 3
No. Layers	3	3	5
ρ_{HA}	0.07± 0.02	0.09± 0.03	0.05± 0.03
z_{HA} [nm]	2.6 ± 0.9	1.3 ± 0.5	1.0 ± 0.5
σ_{HA} [nm]	1.2 ± 0.6	0.8 ± 0.4	0.3 ± 0.1

Table S1 Parameters of the fits of DPPC vesicles with and without HA in sodium chloride solutions. σH/T/HA gives the half width of the Gaussian curve for the head -/ tail group / HA layer, zH/HA describes the distance of the head group/ HA layer from the bilayer center, ρr/HA give the relative electron density of the tail group/ HA layer, d is the d-spacing, n the ratio of uni- to multilamellar structures, A a scaling factor and BG the

without HA	25°C	37°C	50°C
σ_H [nm]	0.22± 0.01	0.45± 0.04	0.32± 0.02
σ_T [nm]	0.40± 0.02	0.48± 0.03	0.48± 0.04
z_H [nm]	2.16± 0.04	2.15± 0.04	1.90± 0.07
ρ_r	0.52± 0.04	1.00± 0.06	1.22± 0.07
d [nm]	-	-	6.0 ± 0.2
n	20 ± 1	19 ± 2	17 ± 2
A	19 ± 2	6.5 ± 0.1	12 ± 1
BG	16 ± 3	25 ± 5	30 ± 4
No. Layers	1	1	3
**with HA**	**25°C w HA**	**37°C w HA**	**50°C w HA**
σ_H [nm]	0.25± 0.01	0.45± 0.02	0.32± 0.03
σ_T [nm]	0.46± 0.03	0.48± 0.04	0.48± 0.04
z_H [nm]	2.20± 0.06	2.15± 0.05	1.95± 0.07
ρ_r	0.52± 0.03	1 ± 0.07	1.22± 0.06
d [nm]	7 ± 0.2	7.3 ± 0.3	6.6 ± 0.2
n	3 ± 0.4	2.9 ± 0.3	5 ± 0.5
A	78± 6	25 ± 2	21 ± 2
BG	45± 5	45 ± 5	44 ± 4
No. Layers	2	2	2
ρ_{HA}	0.08 ± 0.02	0.18 ± 0.05	0.08 ± 0.04
z_{HA} [nm]	1.6 ± 0.6	1.3 ± 0.4	1.5 ± 0.6
σ_{HA} [nm]	0.7 ± 0.3	0.7 ± 0.2	0.6 ± 0.3

Table S2 Parameters of the fits of the DPPC vesicles and DPPC vesicles with and without HA in 150 mM NaCl with 10 mM $CaCl_2$. σH/T/HA gives the half width of the Gaussian curve for the head -/ tail group / HA layer, zH/HA describes the distance of the head group/ HA layer from the bilayer center, ρr/HA give the relative electron density of the tail group/ HA layer, d is the d-spacing, n the ratio of uni- to multilamellar structures, A a scaling factor and BG the background level
